# Prevalence of factors related to active reproductive health behavior: a cross-sectional study Indonesian adolescent

**DOI:** 10.4178/epih.e2016041

**Published:** 2016-09-30

**Authors:** Tantut Susanto, Iis Rahmawati, Emi Wuri Wuryaningsih, Ruka Saito, Syahrul, Rumiko Kimura, Akiko Tsuda, Noriko Tabuchi, Junko Sugama

**Affiliations:** 1 Department of Health Development Nursing, Division of Health Sciences, Graduate School of Medical, Pharmaceutical and Health Sciences, Kanazawa University, Kanazawa, Japan; 2 Family and Community Health Nursing Department, School of Nursing, University of Jember, Jember, Indonesia; 3 Maternity Nursing Department, School of Nursing, University of Jember, Jember, Indonesia; 4 Mental Health Nursing Department, School of Nursing, University of Jember, Jember, Indonesia; 5 Family and Community Health Nursing Department, School of Nursing, Hasanudin University, Makasar, Indonesia; 6 Department of Health Development Nursing, Division of Health Sciences, Institute of Medical, Pharmaceutical and Health Sciences, Kanazawa University, Kanazawa, Japan; 7 Department of Clinical Nursing, Division of Health Sciences, Institute of Medical, Pharmaceutical and Health Sciences, Kanazawa University, Kanazawa, Japan; 8 Wellness Promotion Sciences Center, Institute of Medical, Pharmaceutical and Health Sciences, Kanazawa University, Kanazawa, Japan

**Keywords:** Prevalence, Adolescent behavior, Reproductive health, Culture, Cross-sectional studies

## Abstract

**OBJECTIVES:**

Complex and diverse factors are related to reproductive health (RH) behavior among adolescents according to the social and cultural context of each countries. This study examined the prevalence of active RH and factors related to active RH behavior among Indonesian adolescents.

**METHODS:**

A cross-sectional study was conducted among 1,040 of students who were selected through a multi-stage random sampling technique. A self-administered questionnaire was developed, including the World Health Organization Illustrative Questionnaire for Interview-Surveys with Young People, pubertal development scale, and sexual activity scale, modified in accordance to the Indonesian context. The data were analyzed using descriptive and comparative statistics, as well as logistic regression analyses.

**RESULTS:**

The prevalence of active RH behavior were more higher in boys (56.6%; 95% confidence interval [CI], 50.6% to 62.6%) than in girls (43.7%; 95% CI, 37.6% to 49.8%). Negative attitudes towards RH were a factor related to active RH behavior in both boys and girls. Smoking and kind relationship envisioned before marriage (*pacaran* [courtship] and *nikah siri* [non-registered marriage]) were factors related to active RH behavior in boys; whereas the absence of access to information on substance abuse was an additional factor in girls. Moreover, an interaction was found between access to information on development and smoking (boys) and attitudes on RH (girls) as independent variables associated with active RH behavior.

**CONCLUSIONS:**

Sex education for adolescents in Indonesia, particularly in the context of a health promotion program, should be developed based on prevalent social, cultural, and religious values to prevent active RH behavior. Such programs should focus on the kind of relationship envisioned before marriage and smoking for boys and access to information on subtance abuse for girls.

## INTRODUCTION

Due to the normative standards of Asian countries, especially in the Islamic culture of Indonesia, much debate has emerged about sexual activity and associated factors in adolescents. The culture, ethnicities, and religions of Indonesia differ greatly, and such differences influence adolescents’ reproductive health (RH). Previous studies have reported that Indonesian adolescents confront problems such as early-onset or late-onset puberty; a tendency to delay marriage; sexual activity [1]; and a lack of adequate knowledge, health care, and counseling [2]. However, based on the culture and norms associated with the social environment and religion in Indonesia [3], certain sensitivities and taboos, exist regarding discussing of sexuality and RH [4]. Nonetheless, evidence suggests that although Indonesia is the largest Islamic country to have legal restrictions on pornography, adolescents in Indonesia widely consume pornography [5].

Recently, RH has been recognized as an important health issue among adolescents in Indonesia. The youth population in Indonesia is vulnerable to adolescent health problems. A national survey conducted in Indonesia reported that 10% of girls 15 to 24 years old smoked, 5% drank alcoholic beverages, and 1% used illicit drugs, while boys are more at risk, as 80% smoked, 40% drank alcoholic beverages, and 4% used illicit drugs [6]. Furthermore, 1% of girls and 8% of boys reported having sexual intercourse outside of marriage [6], and 5% of adolescents aged 10 to 24 years have been reported to have engaged in a wide range of behaviors, including behaviors such as masturbation, whereas there are symptoms of premarital sexual behavior, including as intercourse [7]. Meanwhile, secondary sexual development and emotional changes influence dating and the form of risky behavior [8] that Indonesian adolescents call *pacaran* (courtship) [9], as well as influencing the behavior of those who are engaged [10] or in an unregistered marriage, known as *nikah siri* [11]. The Marriage Regulation of the Indonesian government has established the minimum age marriage as 19 years for men and 16 years for women [12]. However, a national survey has shown that 41.9% of women were married between 15 and 19 years of age, while 4.8% were married between 10 and 14 years of age [7]. Encouraging positive adolescent RH behaviors is necessary to improve RH among the youth, and this could be facilitated by the assessment of related factors that influence RH behaviors. In this context, it is essential to describe the health and environment related to adolescent RH behavior during puberty.

Furthermore, taboos and sensitivity exist in Indonesian culture regarding to discussions of sexuality and RH behavior within the family, at school, and in the community. This situation confuses adolescents when they make decisions regarding RH behavior, because the complex environment of social, cultural, and religious life affects the health of adolescents. Adolescents’ RH behavior reflects their rights regarding their sexuality, including their sexual health and RH over the course of their in the lifespan [13]. However, globalization and Western culture are currently influential, bringing freedom and open access to information. This state of affairs provides opportunities for the emergence of active RH behavior, although only limited research has been performed concerning the simultaneous relationships among all these factors in Indonesia. Therefore, the investigation of all relevant variables will clarify the prevalence of factors relating to active RH behavior among adolescents. Thus, the aim of this study was to examine the prevalence of factors relating to active RH behavior in Indonesian adolescents.

## MATERIALS AND METHODS

### Study design

An observational, school-based, cross-sectional study was conducted.

### Setting and sample

This study was conducted in 31 districts of East Java in Indonesia from November 2014 to February 2015. According to national statistical data from 2013, there were 499 junior high schools in these districts (62 schools in urban areas and 437 schools in rural areas), with 164,287 students. In this study, we selected 25% of the total schools. The sample size was estimated using 95% confidence intervals (CIs) and 10% precision. In the first pilot study, the proportion of RH behavior among adolescents was found to be 57% in rural areas and 42% in urban areas. Therefore, the required samples size was found to be 946, with the addition of and additional 10%, resulting in a the total sample size of 1,040. We used a multiple-stage random sampling method to recruit students in this survey. In the preliminary stage, we randomly selected an area (urban or rural), local subdistricts, and schools. At each school, we divided students according to their year of school, and then randomly selected students from each grade of year. The study population consisted of 1,040 students aged 11 to 16 years from 120 schools.

### Ethical considerations

The study was approved by the ethical committee review board of Indonesia (no. 545/H25.1.11/KE/2014). We then obtained ethical and administrative approval from the Department of Political Unity of the Protection of the Public, the National Education District, and the school administrations.

### Instruments

**Figure 1.**
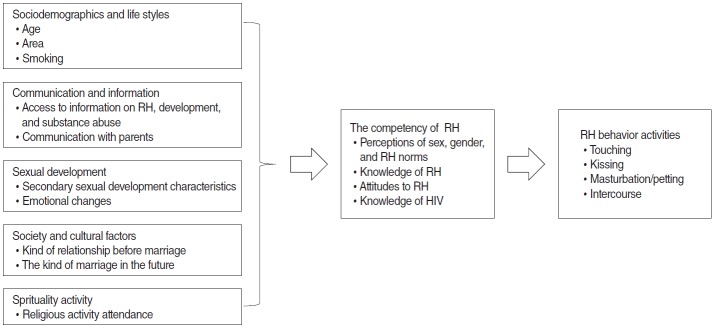
Conceptual schemes of activity reproductive health (RH) behavior among Indonesian adolescents. HIV, human immunodeficiency virus.

We used questionnaires to collect the data. The questionnaire was developed on the basis of the Illustrative Questionnaire for Interview-Surveys with Young People published by the World Health Organization [14]; the Pubertal Development Scale [15]; and the Emotional Changes of Adolescents survey [16] for independent and confounding factors; as well as the sexual activity scale from Bennett and Dickinson’s (1998) Sex Education Inventory [17] for dependent variables modified in accordance with Indonesian society, culture, and religion (Table 1). The variables evaluated in this study are illustrated in Figure 1. The questionnaires used in this study were developed and modified by the researchers after a pilot study and consultation with an expert committee. The expert committee included two professors as student supervisors who were doctoral supervisors of one or more authors. A pilot study was completed to evaluate the validity and reliability of the questionnaires.

### Independent variables

Competency of RH behavior refers to perceptions of sex, gender, and RH norms; knowledge of and attitudes towards RH; and knowledge of human immunodeficiency virus (HIV) [14]. Perceptions of sex, gender, and RH norms reflect the gender norms and rules of adolescents. Perceptions of sex, gender, and RH norms were measured using 21 items using a 3-point Likert-type scale (disagree, 1; neither agree nor disagree, 2; agree, 3). The 21 items were summed to create a composite score of perceptions of sex, gender, and RH norms, with higher scores indicating more positive perceptions of sex, gender, and RH norms (categorized into two groups by median as negative vs. positive).

Participants’ knowledge of RH was assessed using 7 items with yes (1) or no (0) responses. The 7 items were summed to create a composite score of knowledge of RH, with higher scores indicating greater knowledge of RH (categorized into two groups by median as low vs. high). Attitudes to RH reflect how students express their feelings during puberty to parents, teachers, and friends, including how they access information regarding adolescent sexual health and RH in the family, school, and mass media (including pornography that students access during puberty). Attitudes to RH were measured using 11 items (yes, 1; no, 0). The 11 items were summed to create a composite score reflecting attitudes to RH, with higher scores indicating a more positive attitude to RH (categorized into two groups by median as negative vs. positive). Furthermore, knowledge of HIV was assessed using 12 items (yes, 1; no, 0). The 12 items were summed to create a composite score of knowledge of HIV, with higher scores indicating greater knowledge of HIV (categorized into two groups by median as low vs. high).

### Dependent variables

RH behavior was a variable used to measure the activities of adolescents [17]. In this study, participants were given a multiple-response question about their sexual and RH behavior during the period of the study, “What sexual behavior(s) have you engaged in during the period of this study?” with four possible answers (touching, kissing, masturbation and/or petting, or intercourse).

In this study, we categorized RH behavior into two groups (not active and active) based on the Indonesian context. The not active group was categorized as those who reported not engageding in any of the four activities (touching, kissing, masturbation and/or petting, or intercourse), whereas active RH behavior was considered to be any combination of touching, kissing, masturbation/petting, and/or intercourse.

### Confounding variables

The sociodemographic measures included information on the each respondent’s age, gender, area (urban vs. rural), and current smoking behavior (yes or no). We used a modified framework classifying participants into three age groups: early adolescence, 10-14 years; middle adolescence, 15-16 years; and late adolescence, 17-21 years, based on the stages of adolescent development [18]. However, in this study, the age range of participants was 12 to 16 years, so we categorized participants into two groups. Early adolescence was defined as the age range of 12 to 14 years and middle adolescence was defined as the range of 15 to 16 years, although only eight participants were 16 years old. Communication about RH with parents was classified as often, occasionally, or never, and access to information on reproductive health, development, and substance abuse was categorized as access or no access. Additionally, participants were asked what kind of relationship they envisioned before marriage (*pacaran*, engaged, *nikah siri*, or no relationship) and what kind of marriage they planned to have in the future (*nikah siri* vs. legal/governmental). Participants were also asked regarding their attendance of religious services (every day, once per week, or once per month).

Sexual development was a variable used to measure secondary pubertal development and emotional changes that take place during puberty. Secondary pubertal development was measured using the Pubertal Development Scale [15] with six modified items. This questionnaire contains the following 6 items for boys: do they have wet dreams; have they experienced an increase in muscle mass; do they have an Adam’s apple; have they experienced hair growth in their armpits, around the face, and genitalia; do they have more sweaty armpits; and have their voices become deep. The 6 items for girls were: do they menstruate; have theirs breast grown; have their nipples grown; have their hips widened; have they experienced hair growth around the genitalia and in their armpits; and has their skin become more oily (no, 0; yes, 1). The 6 items were summed to create a composite score of secondary sexual development, with higher scores indicating more mature secondary sexual development. Participants were categorized into two groups by by median values as immature vs. mature.

Emotional changes were measured using 4 items assesing psychological and emotional changes during adolescence [16]. The 4 items regarding emotional changes that both genders responded to related to paying more attention to the opposite gender, wishing to be paid attention to, becoming more sensitive, and enjoying looking at oneself in the mirror (no, 0; yes, 1). These 4 items were summed to create a composite score of emotional changes, with higher scores indicating more mature emotional changes. Participants were categorized into two groups by median values as immature vs. mature.

### Data collection

After obtaining consent forms, the investigators distributed a questionnaire to the eligible students. In each of the schools, nine students were selected, with three students invited to participate from each grade. The students filled out the questionnaires in the classrooms. After completing it, the student returned his or her questionnaire to a research investigator. To control for bias, the investigators were nurses and were responsible for guiding the students in filling out the questionnaires at the schools.

### Data analysis

Descriptive statistics (frequencies) were used to calculate the prevalence of RH behavior both boys and girls separately. For univariate analysis, we used the chi-square (χ^2^) or Fisher exact test for categorical data. The cut-off point of p<0.05 was used to select candidate predictors for the multivariate analysis assessing covariance. Furthermore, to determine the factors related with active RH behavior, we used logistic regression analysis to examine relations between active RH behavior in the not active and active groups, as well as independent and confounding variables. Model fit was determined using the Hosmer and Lemeshow test.

We also tested interactions for all covariates to examine significant relationships with active RH behavior in the logistic model to assess the interactions using a multiplicative model, a new variable was created reflecting the interactions independent variables with other independent variables in the model. Further interactions of variables were included in the multivariate analysis, followed by the omnibus test (step) and p-Wald of the interaction variables. If the p-Wald of the interaction variables was ≥0.05, that meant that no interaction was found between the variables, so they were excluded from subsequent analyses. This process was interated until interactions with Wald value as p-value <0.05 were found. All data were analyzed using SPSS version 22 (IBM Corp., Armonk, NY, USA).

## RESULTS

### Prevalence of active reproductive health behavior

A total 1,040 participants were included in this study: 463 boys (44.5%) and 577 girls (55.5%), with a mean age of 13.72 yeras (standard deviation, 0.91 years). In this study, no respondents reported intercourse (active sexual behavior), so we used the three remaining categories of activities (touching, kissing, and masturbation/petting) to classify participants as not active and active according to RH behavior. The not active group was defined as those who reported not engaging in touching, kissing, or masturbation/petting. Active RH behavior was categorized as touching, kissing, and/or masturbation/petting, as well as any combination of these three activities. This was a multiple-response question, which may have influenced the results regarding the prevalence of RH behavior, as presented in Table 2.

Regarding the boys, reported participation in these categories of RH behavior was as follows: touching, 43.0%; kissing, 16.2%; and masturbation, 23.1%. The prevalence of active RH behavior among boys was 56.6% (95% CI, 50.6 to 62.6%). For girls, the prevalent RH behaviors were touching (36.2%), kissing (10.7%), and masturbation (6.6%). Therefore, the prevalence of active RH behavior among girls was 43.7% (95% CI, 37.6 to 49.8%).

### Factors related to active reproductive health behavior among boys

For the univariate analysis, we evaluated candidate factors for potentially related to the presence of active RH behavior among boys, with p-value <0.05 (Table 3). After controlling for all other covariates, multiple logistic regression analysis (Table 4) showed that the factors related to active RH behavior among boys were smoking (yes) OR, 5.13; 95% CI, 1.98 to 13.31, absence of access to information on development OR, 3.12; 95% CI, 1.76 to 5.51, the kind relationship envisined before marriage being *pacaran* OR, 3.79; 95% CI, 1.64 to 8.76, or *nikah siri* OR, 5.92; 95% CI, 1.79 to 19.63, and negative attitudes regarding RH OR, 8.98; 95% CI, 5.57 to 14.50.

In the analysis of all covariates for interactions, we found interaction effects between smoking and access to information on development among boys. A combined OR of 0.16 (95% CI, 0.03 to 0.76) was found, although the precise nature of the interaction remains unclear.

### Factors related factors to active reproductive health behavior among girls

The factors significantly associated with active RH behavior among girls are presented in Table 3. After controlling for all other covariates, multiple logistic regression analysis (Table 4) showed that the factors associated with active RH behavior among girls were the absence of access to information on development OR, 3.36; 95% CI, 1.84 to 6.15 and substance abuse OR, 2.97; 95% CI, 1.69 to 5.27, as well as negative attitudes regarding RH OR, 10.85; 95% CI, 7.03 to 16.72.

In the analysis of all covariates for interactions, we found interaction effects between access to information on development and negative attitudes regarding RH among girls. A combined OR, 0.22; 95% CI, 0.08 to 0.60 was found, although the precise nature of the interactions remains unclear.

## DISCUSSION

### Prevalence of active reproductive health behavior among boys and girls

The current study found that the prevalence of active RH behavior among boys and girls was 56.6% and 43.7%, respectively. These results are high compared to the sexual behavior of adolescents in the city of Pontianak, Indonesia (19.2%) [19], although they are lower than levels reported in Malaysia (55.1%) [20]. This difference in results may at least partially reflect differences in the age range of participants (13 to 15 years old in Pontianak and 12 to 19 years old in Malaysia); age is associated with the development of sexual maturity and its effect on sexual behavior, although these studies were conducted in similar sociocultural backgrounds. Regarding the activities defined as active RH behavior, the percentage of respondents who reported touching (39.2%), kissing (13.3%), and masturbation (13.9%) in this study is higher than has been found in previous studies in Indonesia (kissing, 5.5%; necking, 3.5%; and petting, 2.7%) [19], although it still remains lower than has been found in the Islamic culture of Turkey (masturbation, 53.3%) [21] and the Western country of Norway (kissing, 84.9% and masturbation, 64.1%) [22]. This may indicate that adolescents develop sexual behavior according to the stage of their sexual development.

Unexpectedly, intercourse was not reported in this study. These findings differ from previous studies showing that 13% of adolescent men and 2% of adolescent women engaged in sexual intercourse in Indonesia [23], whereas 5.4% of adolescent Cambodians [24] and 2.3% of adolescent Malaysians [25] had participated in premarital sexual intercourse. These discrepancies may be explained by the fact that the participants mostly lived in a rural area, with limited access to information, corresponding to a slow influence of Western culture on RH behavior. The results of this study indicated that the prevalence of active RH behavior was the lowest in the Indonesian context. However, it is likely to increase in association with modernization and Westernization [3], leading to more access to information on RH in Indonesia [23]. Thus, our findings suggest the importance of health-promotion strategies for youth to prevent active RH behavior in Indonesia.

### Factors related to active reproductive health behavior among boys

Four factors were related to active RH behavior among boys: smoking, access to information on development, the type of relationship envisioned before marriage, and attitudes about RH. In this study, smoking was related with active RH behavior among boys, which is consistent with a previous study in Malaysia [20]. This finding might be explained by the fact that adolescents attracted to new and challenging risky experiences, and that life problems during puberty associated with behavioral problems are also associated with adolescent smoking. Cigarette smoking may lead to behavioral problems, which in turn may influence adolescents to engage in RH behavior. This may provide insight into the main challenges in developing and implementing health education in Indonesia among adolescents, in particular the need to prevent smoking and its potential influence on RH behavior.

We also found that the absence of access to information on development was related to active RH behavior among boys. This finding is consistent with previous studies reporting that sources of information [26] are associated with RH behavior. This might be explained by the fact that boys are in need of information on the physical, psychological, and emotional phases of growth and development in order to adapt to changes during puberty. These results emphasize the importance of providing sources of correct information and promoting knowledge and understanding of RH issues among adolescents in Indonesia in order to encourage positive attitudes regarding sexual topics, based on the social and cultural context of Indonesia.

Our findings that envisioning *pacaran* and *nikah siri* as types of relationships before marriage were related with active RH behavior in boys are similar to those of a previous study in Indonesia [27,28]. This indicates that puberty and RH maturation may cause sexual urges in adolescents who begin to experiment with sexuality through *pacaran*. This relationship was found among adolescents who were drawn to express feelings through active RH behavior, although this is traditionally the period in which individuals transition towards the age of marriage in Indonesia [29]. These patterns may illustrate a limited degree of RH self-awareness regarding gender and social norms during puberty. This suggests that the age of marriage should be increased to prevent premarital sexual activity among adolescents in Indonesia.

Moreover, we found that attitudes towards RH were associated with a greater likelihood of active RH behavior among boys. These findings are consistent with previous studies in India [30]. This may be associated with the acceptance of certain gender roles, since adolescent RH behavior develops in accordance with gender and environmental norms. This suggests that Indonesian adolescents need sex education programs to improve their knowledge, attitudes, and behaviors, as well as to encourage the formation of positive attitudes regarding gender and RH norms.

### Factors related to active reproductive health behavior among girls

In this study, access to information on development and substance abuse, as well as attitudes on RH, were found to be associated with active RH behavior in girls. In particular, the absence of access to information on development was related to active RH behavior among girls. The explanation for these findings may be similar to that proposed for boys. Therefore, the provision of more accessible to information on development may help adolescents to develop more positive RH behavior. Changes in regulations relating to access to adolescent health information through mass media and the internet may also help to increase healthy RH behavior.

Our findings showed that girls who did not have adequate access to information on substance abuse were more likely to engage in active RH behavior, which is similar to the findings of previous studies that pubertal timing may also be associated with an elevated risk of substance abuse [31,32]. This may be explained by the fact that girls show an earlier onset of puberty and by the complexity of woman secondary sexual development, which may cause complex range of challenges for girls. Therefore, girls need focused access to substance abuse information through educational programs. These findings suggest that it would be desirable to develop infrastructure for the communication of information and socialization regarding the danger of substance abuse during puberty through channels that adolescents may access through school health programs, and family. In addition, attitudes on RH were associated with active RH behavior among girls. These findings are consistent with previous studies for the same reason that was proposed regarding the findings among boys.

In this study, logistic regression analysis showed interactions between independent variables as factors associated with active RH behavior in both boys and girls. Among boys, it was unclear whether current smoking may have been related to active RH behavior based on access to information on development or vice versa. These findings are similar to those of previous studies that found that smoking and information sources [20] were associated with RH [33]. In contrast, among girls, it was not clear whether access to information on development was related to active RH behavior as mediated by attitudes to RH or vice versa. This findings are similar to those of previous studies reporting that access to information [25] and pubertal development [33] were related to attitudes regarding gender and norms. This may be explained by the availability of resources for the development of adolescent health, and these findings suggest the need to provide health education about the dangers of smoking during adolescent development to minimize risky behavior and encourage positive, balanced attitudes regarding gender and RH norms during puberty based on the social and cultural context of Indonesia.

The finding of the current study that intercourse (active sexual behavior) was not reported may reflect the fact that sexuality and adolescent RH are sensitive issues in Indonesian culture and society, which may have influenced our data collection. However, in this study, in order to maximize the accuracy of the responses, participants were first asked to respond to questions about their sociodemographic characteristics, communication patterns, information about RH, and competency of RH, and only then were participants encouraged to complete the questionnaire about sexual development, society and culture, spirituality, and RH behavior in a private room. This suggests that future qualitative research could assess the phenomenon of intercourse among adolescents with appropiate cultural sensitivity in the Indonesian context.

This study has limitations, including the cross-sectional nature of the study design, which resulted in associations being found among the variables, rather than causal conclusions. In this study, the CIs were very wide, which casts doubt upon the certainty of the effects, and the fact that further information is needed reflects the sample size. Furthermore, this is a limitation regarding the measured prevalence of active RH behavior in this study, which may be different from what has been found in other studies, based on definitions of RH behavior and sample size.

The current study showed that access to information on development and negative attitudes towards RH were factors associated with active RH behavior in both boys and girls. Meanwhile, current smoking habit and envisioning *pacaran* before marriage were associated with active RH behavior in boys, whereas access to information on substance abuse was an additional factor associated with active RH behavior in girls. Thus, the study results suggest that health-promotion programs on sexual health and RH could be designed to provide accurate information grounded in the social, environmental, and religious aspects of the Indonesian context, focusing the content of sex education on concerns regarding adolescent development in order to prevent the development of negative attitudes regarding RH. Health promotion programs should be designed to focus on smoking and preventing relationships before marriage for boys and the provision of information on substance abuse for girls in the Indonesian context.

## Figures and Tables

**Table 1.** Variables investigated in the current study

Elements of RH	Variable	Measures [Ref]
Sociodemographics informations	Age and gender	Sociodemographic questionnaire
	Area	
	Current smoking	
Sources of information and communication	Access to information on RH	Illustrative Questionnaire for Interview-Surveys with Young People, WHO [14]
	Access to information on development	
	Access to information on substance abuse	
	Communication about RH with parents	
Spirituality	Attending religious services	Illustrative Questionnaire for Interview-Surveys with Young People, WHO [14]
Sexual development	Secondary sexual development	Pubertal Development Scale [15]
	Emotional changes	Psychological symptoms [16]
Social and cultural	Kind of relationship before marriage	Questionnaire regarding youth relationships in Indonesian culture
	Kind of marriage in the future	
Competencies of RH	Knowledge of RH	Illustrative Questionnaire for Interview-Surveys with Young People, WHO [14]
	Knowledge of HIV	
	Attitudes on RH	
	Perceptions of sex, gender, and RH norms	
RH behavior	Touching	Sexual activity scale from Bennett and Dickinson (1998) and Sex Education Inventory [17]
	Kissing	
	Petting and/or maturbation	
	Intercourse	

RH, reproductive health; HIV, human immunodeficiency virus; WHO, World Health Organization.

**Table 2.** Prevalence^1^ of active reproductive health (RH) behaviors among boys and girls (n=1,040)

RH behaviors	Total	Boys	Girls
Not active^2^	514 (49.4) [20.5, 31.0]	201 (43.3) [36.6, 50.3]	325 (56.3) [50.9, 61.7]
Active^3^	526 (50.6) [46.3, 54.8]	262 (56.6) [50.6, 62.6]	252 (43.7) [37.6, 49.8]
Touching			
Yes	408 (39.2) [34.5, 44.0]	199 (43.0) [36.1, 49.9]	209 (36.2) [36.1, 36.3]
No	632 (60.8) [57.0, 64.6]	264 (57.0) [51.0, 63.0]	368 (63.8) [58.9, 68.7]
Kissing			
Yes	137 (13.2) [7.51, 18.8]	75 (16.2) [7.9, 24.5]	62 (10.7) [3.0, 18.5]
No	903 (86.8) [84.6, 89.0]	388 (83.8) [80.1, 87.5]	515 (89.3) [86.6, 91.9]
Masturbation			
Yes	145 (13.9) [8.3, 19.6]	107 (23.1) [15.1, 31.1]	38 (6.6) [-1.3, 14.5]
No	895 (86.1) [83.8, 88.3]	356 (76.9) [72.5, 81.3]	539 (93.4) [91.3, 95.5]

Values are presented as number (%) and [95% confidence interval].

RH, reproductive health; CI, confidence interval.

1 Participants could choose more than one response; Respondents’ replies were assessed as not active or active based on the Indonesian context.

2 Not active RH behavior was defined as no practice of any of 3 activities (touching, kissing, or masturbation).

3 Active RH behavior was defined as engaging in any of those 3 activities (touching, kissing, or masturbation).

**Table 3.** Responses regarding sociodemographic, communication and information, religious behavior, pubertal development, and social and cultural, competencies of RH by RH activity (n=1,040)

RH activity	Boys			p-value^1^	Girls			p-value^1^
	Total	Not active	Active		Total	Not active	Active	
Age (yr)								
12-14	173 (37.4)	83 (48.0)	90 (52.0)	0.15	234 (40.6)	139 (59.4)	95 (40.6)	0.25
15-16	290 (62.6)	118 (40.7)	172 (59.3)		343 (59.4)	186 (54.2)	157 (45.8)	
Area								
Urban	152 (32.8)	72 (47.4)	80 (52.6)	0.27	180 (31.2)	108 (60.0)	72 (40.0)	0.27
Rural	311 (67.2)	129 (41.5)	182 (58.5)		397 (68.8)	217 (54.7)	180 (45.3)	
Smoking								
Yes	66 (14.3)	12 (18.2)	54 (81.8)	<0.001	9 (1.6)	3 (33.3)	6 (66.7)	0.29
No	397 (85.7)	189 (47.6)	208 (52.4)		568 (98.4)	322 (56.7)	246 (43.3)	
Access to information on RH								
No	130 (28.1)	45 (34.6)	85 (65.4)	0.02	90 (15.6)	44 (48.9)	46 (51.1)	0.15
Yes	333 (71.9)	156 (46.8)	177 (53.2)		487 (84.4)	281 (57.7)	206 (42.3)	
Access to information on development								
No	118 (25.5)	33 (28.0)	85 (72.0)	<0.001	95 (16.5)	40 (42.1)	55 (57.9)	0.003
Yes	345 (74.5)	168 (48.7)	177 (51.3)		482 (83.5)	285 (59.1)	197 (40.9)	
Access to information on substance abuse								
No	86 (18.6)	29 (33.7)	57 (66.3)	0.06	80 (13.9)	28 (35.0)	52 (65.0)	< 0.001
Yes	377 (81.4)	172 (45.6)	205 (54.4)		497 (86.1)	297 (59.8)	200 (40.2)	
Communication about RH with parents								
Often	47 (10.2)	17 (36.2)	30 (63.8)	0.02	129 (22.4)	71 (55.0)	58 (45.0)	0.03
Occasionally	207 (44.7)	78 (37.7)	129 (62.3)		332 (57.5)	176 (53.0)	156 (47.0)	
Never	209 (45.1)	106 (50.7)	103 (49.3)		116 (20.1)	78 (67.2)	38 (32.8)	
Attendance at religious services								
Every day	310 (67.0)	138 (44.5)	172 (55.5)	0.001	406 (70.4)	224 (55.2)	182 (44.8)	0.24
Once per week	97 (21.0)	51 (52.6)	46 (47.4)		125 (21.7)	78 (62.4)	47 (37.6)	
Once per month	56 (12.1)	12 (21.4)	44 (78.6)		46 (8.0)	23 (50.0)	23 (50.0)	
Secondary sexual development								
Immature	166 (35.9)	83 (50.0)	83 (50.0)	0.04	252 (43.7)	153 (60.7)	99 (39.3)	0.07
Mature	297 (64.1)	118 (39.7)	179 (60.3)		325 (56.3)	172 (52.9)	153 (47.1)	
Emotional changes								
Immature	165 (35.6)	88 (53.3)	77 (46.7)	0.002	148 (25.6)	97 (65.5)	51 (34.5)	0.01
Mature	298 (64.4)	113 (37.9)	185 (62.1)		429 (74.4)	228 (53.1)	201 (46.9)	
Kind of relationship before marriage								
*Pacaran*^2^	139 (30.0)	51 (36.7)	88 (63.3)	<0.001	146 (25.3)	85 (58.2)	61 (41.8)	0.67
Engaged	237 (51.2)	107 (45.1)	130 (54.9)		340 (58.9)	187 (55.0)	153 (45.0)	
*Nikah siri*^3^	39 (8.4)	7 (17.9)	32 (82.1)		40 (6.9)	21 (52.5)	19 (47.5)	
No relationships	48 (10.4)	36 (75.0)	12 (25.0)		51 (8.8)	32 (62.7)	19 (37.3)	
Kind of marriage in the future								
*Nikah siri*^3^	45 (9.7)	10 (22.2)	35 (77.8)	0.004	38 (6.6)	16 (42.1)	22 (57.9)	0.10
Legal/governmental	418 (90.3)	191 (45.7)	227 (54.3)		539 (93.4)	309 (57.3)	230 (42.7)	
Perceptions of sex, gender, and RH norms								
Negative	280 (60.5)	114 (40.7)	166 (59.3)	0.18	268 (46.4)	151 (56.3)	117 (43.7)	1.00
Positive	183 (39.5)	87 (47.5)	96 (52.5)		309 (53.6)	174 (56.3)	135 (43.7)	
Knowledge of RH								
Low	190 (41.0)	97 (51.1)	93 (48.9)	0.008	272 (47.1)	142 (52.2)	130 (47.8)	0.07
High	273 (59.0)	104 (38.1)	169 (61.9)		305 (52.9)	183 (60.0)	122 (40.0)	
Knowledge of HIV								
Low	269 (58.1)	128 (47.6)	141 (52.4)	0.04	355 (61.5)	204 (57.5)	151 (42.5)	0.54
High	194 (41.9)	73 (37.6)	121 (62.4)		222 (38.5)	121 (54.5)	101 (45.5)	
Attitudes on RH								
Positive	254 (54.9)	166 (65.4)	88 (34.6)	<0.001	347 (60.1)	259 (74.6)	88 (25.4)	< 0.001
Negative	209 (45.1)	35 (16.7)	174 (83.3)		230 (39.9)	66 (28.7)	164 (71.3)	

Values are presented as number (%).

RH, reproductive health; HIV, human immunodeficiency virus.

1 p-values are obtained by chi-square or Fisher exact test.

2 *Pacaran* is term referring to a common pattern of courtship between boys and girls in Indonesia.

3 *Nikah siri* is marriage that is unregistered with the government because it is not reported to the Office of Religious Affairs (for Muslims) or the Civil Registry Office (for non-Muslims) in Indonesia.

**Table 4.** Logistic regression analysis of factors related to active reproductive health (RH) behavior among boys and girls (n=1,040)

Variable	Boys^1^	Girls^2^
Smoking		
No	1.00	
Yes	5.13 (1.98, 13.31)^**^	
Access to information on development		
Yes	1.00	
No	3.12 (1.76, 5.51)^***^	3.36 (1.84, 6.61)^***^
Access to information on substance abuse		
Yes	1.00	
No	-	2.97 (1.69, 5.27)^***^
Kind of relationship before marriage		
No relationship	1.00	
*Pacaran*^3^	3.79 (1.64, 8.76)^**^	
Engaged	2.07 (0.93, 4.59)	
*Nikah siri*^4^	5.92 (1.79, 19.63)^**^	
Attitudes on RH		
Positive	1.00	
Negative	8.98 (5.57, 14.50)^***^	10.85 (7.03, 16.72)^***^
Access to information on developmentx smoking		
Yes x no smoking^5^	0.16 (0.03, 0.76)^*^	
No x smoking		
Access to information on developmentx attitudes on RH		
Yes x positive^5^		0.22 (0.08, 0.60)^*^
No x negative		

Values are presented as odds ratio (95% confidence interval).

1 For boys: -2LLχ^2^=468.72 (p<0.001); Hosmer and Lemeshow test (χ^2^)=4.62 (p=0.71); Cox and Snell R^2^=0.30; Nagelkerke R^2^=0.40.

2 For girls: -2LLχ^2^=629.29 (p<0.001); Hosmer and Lemeshow test (χ^2^)=1.45 (p=0.69); Cox and Snell R^2^=0.24; Nagelkerke R^2^=0.33.

3 *Pacaran* is term referring to a common pattern of courtship between boys and girls in Indonesia.

4 *Nikah siri* is marriage that is unregistered with the government because it is not reported to the Office of Religious Affairs (for Muslims) or the Civil Registry Office (for non-Muslims) in Indonesia.

5 Interaction of both independent variables.

* p<0.05;

** p<0.01;

*** p<0.001.
